# Renewing the respect for similarity

**DOI:** 10.3389/fncom.2012.00045

**Published:** 2012-07-13

**Authors:** Shimon Edelman, Reza Shahbazi

**Affiliations:** Department of Psychology, Cornell University, IthacaNY, USA

**Keywords:** object recognition, scene interpretation, scene space, shape space, similarity, view space, visual structure

## Abstract

In psychology, the concept of similarity has traditionally evoked a mixture of respect, stemming from its ubiquity and intuitive appeal, and concern, due to its dependence on the framing of the problem at hand and on its context. We argue for a renewed focus on similarity as an explanatory concept, by surveying established results and new developments in the theory and methods of similarity-preserving associative lookup and dimensionality reduction—critical components of many cognitive functions, as well as of intelligent data management in computer vision. We focus in particular on the growing family of algorithms that support associative memory by performing hashing that respects local similarity, and on the uses of similarity in representing structured objects and scenes. Insofar as these similarity-based ideas and methods are useful in cognitive modeling and in AI applications, they should be included in the core conceptual toolkit of computational neuroscience. In support of this stance, the present paper (1) offers a discussion of conceptual, mathematical, computational, and empirical aspects of similarity, as applied to the problems of visual object and scene representation, recognition, and interpretation, (2) mentions some key computational problems arising in attempts to put similarity to use, along with their possible solutions, (3) briefly states a previously developed similarity-based framework for visual object representation, the Chorus of Prototypes, along with the empirical support it enjoys, (4) presents new mathematical insights into the effectiveness of this framework, derived from its relationship to locality-sensitive hashing (LSH) and to concomitant statistics, (5) introduces a new model, the Chorus of Relational Descriptors (ChoRD), that extends this framework to scene representation and interpretation, (6) describes its implementation and testing, and finally (7) suggests possible directions in which the present research program can be extended in the future.

## 1. The ubiquity of similarity

The effectiveness of an embodied cognitive system in fending for itself depends on its ability to gain insights into its situation that may not be immediately obvious, either because the properties of interest are not explicit in its sensory assessment of the outside world, or, more interestingly, because they are projections into a potential future. Species that share an ecological niche cannot entirely avoid the need for forethought, or reasoning about the future (Dewey, 1910; Craik, 1943; Dennett, 2003; Edelman, 2008; Bar, 2011). Indeed, evolutionary experiments in which a species seemingly drops out of the smarts race by opting for thicker armor or bigger teeth are merely bets that these bodily attributes will continue to be effective in the future. Such bets that are likely to go horribly wrong when a competitor invents the next brainy countermeasure to brawn.

Forethought works because the world is “well-behaved” in the sense that the future *resembles* the remembered past and can be often enough estimated from it, in relevant respects, and up to a point. In particular, similar consequences are likely to follow from similar observable causes—an observation that has influenced philosophical thought since Aristotle and that has been expressed forcefully by Hume (1748, ch. IX):

ALL our reasonings concerning matter of fact are founded on a species of Analogy, which leads us to expect from any cause the same events, which we have observed to result from similar causes. Where the causes are entirely similar, the analogy is perfect, and the inference, drawn from it, is regarded as certain and conclusive. […] But where the objects have not so exact a similarity, the analogy is less perfect, and the inference is less conclusive; though still it has some force, in proportion to the degree of similarity and resemblance.

While Hume's observation applies to visual objects and scenes just as it does to all of cognition, bringing out similarity in vision and putting it to work requires some extra ingenuity on the part of any visual system, natural or artificial. In particular, to obtain information regarding the *shapes* of the objects that are present in the scene, the visual system must overcome the effects of the orientation of objects, of their juxtaposition, and of illumination. As it turns out that these computational challenges are subsumed under the general rubric of similarity-based processing, we shall begin by considering the most general issues first^1^.

The past several decades saw a concerted effort to put the explanatory role of similarity in psychology on a mathematical foundation. One well-known approach has employed set-theoretical tools (Tversky, 1977; Tversky and Gati, 1978); another one resulted in the development, from first principles, of a theory of similarity based on metric representation spaces (Shepard, 1980, 1984, 1987). In the present brief overview, we initially focus on the metric-space approach (although, as we shall see, the differences between the two turn out to be immaterial).

The basic premise of the metric theory of similarity posits that a perceiver encodes entities that are of interest to it, such as visual objects, scenes, or events, as points in a representation space in which perceived similarity between two items is monotonically related to their proximity. Shepard (1987) showed that a few fundamental assumptions, such as the Bayes theorem and the maximum entropy principle, lead to a representation space endowed with the Minkowski *l*_*p*_ metric (with *p* = 1 if its dimensions are separable (Attneave, 1950; Garner and Felfoldy, 1970) and *p* = 2 if they are not), and that the dependence of generalization from one item to another on their similarity—that is, on the representation-space distance—is negative exponential.

This dependence of generalization on representation-space distance had been found to hold for a range of taxa and tasks, from hue discrimination in goldfish to vowel categorization in humans. Shepard (1987) interpreted the ubiquity of this pattern as evidence for a universal law of generalization. This idea has been revisited in a special issue of the *Behavioral and Brain Sciences* (Shepard, 2001), where it has also been given a Bayesian formulation (Tenenbaum and Griffiths, 2001). Its empirical support has also been broadened. In a typical study, a confusion table for a set of stimuli is first formed by measuring same/different error rates for each pair of stimuli (this can be accomplished by various means; cf. Cutzu and Edelman, 1998). The table is then submitted to multidimensional scaling (MDS; Beals et al., 1968; Shepard, 1980), which yields a spatial configuration of the stimuli in a metric space of prescribed dimensionality (usually two or three) that best fits the confusion table data. Finally, the probability of generalization is plotted against distance in this “psychological space,” invariably resulting in a negative exponential dependence.

Chater and Vitányi (2003) have recently shown that this dependence of generalization on similarity must hold in principle even without the assumption that items are represented by points in a Minkowski metric space. Resorting instead to the notion of algorithmic information distance, defined as the length of the shortest program that transforms the representations of the two items that are being compared into one another, Chater and Vitányi derived the same negative exponential dependence as in Shepard's formulation. They also noted that their “generalized law of generalization” holds even for “complex visual or linguistic material that seems unlikely to embed naturally into a multidimensional psychological space.”

Combined with the assumption that the world is well-behaved in the sense that similar situations occur often enough and have similar consequences, Shepard's Universal Law of generalization suggests that cognitive processes that guide behavior all conform to the same functional template. A cognitive system faced with a potentially novel situation needs (1) to determine where the new representation lands in the space of prior experience, (2) to look up records of the consequences of responses to similar situations, (3) to use those in thinking ahead to likely outcomes of possible responses, and (4) to generate an actual response while taking into account these data. Notably, this functional template applies all across cognition, from perception (as when conceptual knowledge is distilled from similar pieces of episodic information) to thinking (as in case-based reasoning) and action (where behavioral plans and motor programs are synthesized from whatever worked in the past).

In the remainder of this paper, we offer a series of discussions highlighting a series of conceptual, mathematical, computational, and empirical aspects of similarity, as applied to the problems of visual object and scene representation, recognition, and interpretation. Section 2 discusses certain issues with similarity and argues that these need not prevent it from being a useful explanatory concept in cognition. Sections 3 and 4 offer, respectively, a very brief introduction to a similarity-based framework for visual object representation, the Chorus of Prototypes, and an equally brief overview of the empirical support it enjoys (with multiple references to a detailed treatment elsewhere). In section 5, we present some new mathematical insights into the effectiveness of this framework, derived from its relationship to locality-sensitive hashing (LSH) and to concomitant statistics. Section 6 introduces a new model, the Chorus of Relational Descriptors (ChoRD), that extends this framework to scene representation and interpretation. An implementation and testing of the ChoRD model is described in section 7. Finally, section 8 offers some conclusions and suggests possible directions in which the present research program can be extended in the future.

## 2. The problematicity of similarity

Although first-principles considerations of the kind invoked by Shepard (1987), Tenenbaum and Griffiths (2001), and Chater and Vitányi (2003) clearly suggest that similarity should serve as an indispensable and broad foundation for cognition, its status as an explanatory concept in psychology and in neuroscience has been subject to much doubt (Goodman, 1972; Tversky, 1977; Tversky and Gati, 1978; Rips, 1989; Medin et al., 1993; Townsend and Thomas, 1993; Hahn and Chater, 1998). The prime reason for this is the ambiguity of similarity with regard to items that vary along independent or potentially conflicting dimensions.

Any two objects or situations that are not identical to each other are bound to be similar in some respects and dissimilar in others. As Eisler (1960, p. 77) put it, “An observer instructed to estimate the similarity of e.g., two differently colored weights, is supposed to ask: in what respect?” Because the *respects* in which objects are to be compared do generally depend on the task and on the mindset that the subject brings to it, similarity appears to be too ill-defined to have explanatory value for the psychologist or, indeed, practical value for the perceiver.

This conceptual difficulty is, however, not insurmountable. Rather than seeking an ironclad, universally valid set of similarity relations that are prior to any experience, cognitive systems use their experience in interacting with the world to learn the respects in which various situations should be considered as similar, by tracking the *consequences* of their actions. The similarity question thus turns out to be an instance of the well-known computational problem of credit assignment (Minsky, 1961). Here, it takes the form of the need to differentiate between those features (dimensions) of similarity of two items that are, in the context of the task, predictive of the consequences of generalizing between them, and those that are not^2^.

In general, the credit assignment problem has both temporal (diachronic) and structural aspects. The former has to do with apportioning credit to each of a potentially long sequence of actions, and the latter—to the various dimensions of the situation/action representation. With regard to similarity-based processing, it is the dimensionality of the representation space that is of prime concern. The three related computational problems discussed below all arise from the typically *high dimensionality* of measurement and representation spaces.

The need for high-dimensional representation spaces in cognition stems in turn from the foundational role of experience in the planning of future behavior. To increase the chances that at least some of the stored data would bring out the similarity patterns on which generalization can be based, an advanced cognitive system must measure up as many episodes of its interaction with the world as possible, while making each measurement as detailed as possible. It is no wonder, then, that the amount of information that the brains of long-lived animals in complex ecosystems must capture, process, and store is vast (Merker, 2004). To understand how the brains of such animals, including ourselves, manage this deluge of data, we must first identify the computational principles that are in the play.

### 2.1. The tug of war between content-based retrieval and generalization

Seeing that storage as such appears to be cheap (e.g., Brady et al., 2008), the main problem here is retrieval. In other words, if a vast amount of data is stored against a possible future need, the efficiency of retrieval becomes all the more important. Clearly, retrieval must be selective: only those records that are similar to the present experience must be brought to the fore. Moreover, retrieval must be fast: a sequential scan of the full contents of the multitude of stored items will not do. A computational scheme that fulfills these requirements is *hashing* (Aho et al., 1974). By storing each item under a key that is computed from its content and that uniquely specifies a memory address, hashing allows fast associative recall: a test item can be looked up in constant time, independent of the number of stored items. In that respect, hashing is like a massively parallel, content-addressable biological memory system, in which a cue can be compared simultaneously to multiple stored items (see Willshaw et al., 1969 for an early computational model and Lamdan and Wolfson, 1988 for an early application in a computer vision system for object recognition).

To minimize recall mistakes stemming from memory collisions, hashing functions in data management applications were traditionally engineered to map any two items, even similar ones, to very different addresses. This way, the probability of confusing distinct items could be kept low—but only at the expense of destroying any similarity relationships that may hold over the items. Because under a classical hashing scheme two similar and therefore possibly related cues may wind up very far apart in the representation space, simply “looking around” the address of the best-matching item for anything that may be worth retrieving along with it would not work. Thus, while enabling content-based retrieval, classical hashing hinders similarity-based generalization.

### 2.2. The challenge of dimensionality reduction

Earlier in this section we noted that the measurement space in which objects external to the system are first represented is likely to be high-dimensional. Indeed, in the human visual system, the nominal dimensionality of the input signal from each eye is equal to the number of axons that comprise the optic nerve, or about 10^6^. Any perceivable similarities over visual objects or scenes must, therefore, exist as patterns in that multidimensional signal^3^. The task of finding such patterns is, however, extremely hard.

What kind of measurement-space pattern could be useful for similarity-based generalization? Two generic types of patterns are those that afford categorization and those that support regression (Edelman and Intrator, 2002; Bishop, 2006). In the first case, a number of previously encountered exemplars fall into a small number of distinct categories according to some characteristics, making it possible to categorize a new item by its similarity to each of those. In the second case, exemplars cluster in a subspace of dimensionality that is lower than that of the original measurement space. In each of the two cases, subsequent generalization becomes possible because the description of the data in terms of the patterns is simpler than the original representation (as per the Minimum Description Length (MDL) principle; cf. Adriaans and Vitányi, 2007).

The problem is that the characteristics that define the “small number” of clusters or the “lower-dimensional” subspace in the above formulation need not correspond to any of the original measurement dimensions by themselves. The similarity of two spatially sampled visual objects, for instance, is always distributed over a multitude of pixels (that is, dimensions) rather than being confined to a single pixel. The visual system must find the right function of pixel values (e.g., a rotation of the original space followed by a projection onto a subspace, if the function is constrained to be linear) under which the sought-after similarity pattern—in the two-category case, a bimodal distribution—is made explicit (in the sense of Marr, 1982).

The linear version of the problem of finding such a function is known as projection pursuit (Huber, 1985). By the central limit theorem, most low-dimensional projections of a high-dimensional “cloud” of points will be approximately normal, that is, they will look like noise. Consequently, an “interesting” projection is one that yields a distribution that deviates from normality, e.g., because it is bimodal, or perhaps heavy-tailed (Intrator and Cooper, 1992). Algorithms based on this approach can be extremely effective in cases where the pattern of interest is indeed linear (e.g., two linearly separable clusters of data points side by side). They are, however, of no avail in the general case, where no linear projection can do the job (e.g., if the pattern consists of two concentric spherical shells of data points).

### 2.3. The complexity of learning from examples

A complementary problem to the separation of a pattern into a few clusters or a subspace of a few dimensions is that of pattern build-up. How many data points suffice to define a pattern that can support reliable generalization? This question is of central concern in machine learning (along with the related issue of the number of degrees of freedom of the learning mechanism; e.g., Haussler, 1992). Intuitively, learning from examples can be seen as an instance of function approximation (Poggio, 1990), which suggests that the set of examples must cover the domain of the sought-after function in a representative manner^4^.

The need to cover the representation space with examples implies that the number of required data points depends exponentially on the number of dimensions of the representation space—a problem known as the curse of dimensionality (Bellman, 1961). While it can be circumvented in supervised learning on a task-by-task basis^5^, the problem of dimensionality in an exploratory (unsupervised) setting or in a situation where transfer of performance is expected between tasks (Intrator and Edelman, 1996) must be addressed by undertaking dimensionality reduction prior to learning.

### 2.4. The truth is out there

The last computational consideration that we would like to bring to bear on the problem of learning and use of similarity is that perceptual similarity (as opposed to arbitrary associations that the cognitive system may form following experience) is “out there” in the world, waiting to be transduced into the measurement space and preserved and discovered in the reduced-dimensionality representation. In the domain of visual object shapes, for instance, natural similarity relations arise from the mathematics of shape parametrization, where certain uniqueness results have been proved (see Edelman, 1999, App.C for references). As noted in the introduction, these relations are in principle discoverable by agents situated in the world, insofar as similar causes tend to lead to similar consequences.

This observation suggests that perceptual representations should be evaluated on the basis of their *veridi-cality*—the degree to which they preserve the qualities of the objects “out there.” In particular, a veridical representation scheme that preserves relational qualities such as similarity amounts to what Shepard (1987, 2001; cf. Shepard and Chipman, 1970) termed a second-order isomorphism between the representations and their targets (this must be distinguished from first-order isomorphism, which posits representations that individually resemble their respective objects and which, it should be noted, merely postpones the problem of making sense of the world rather than solving it; Edelman, 1999)^6^.

We may therefore conclude that the twofold computational challenge that any perceptual system must address is (1) to achieve veridical representation of similarities among objects, so as to forge a link between sensory data and consequentially responsible behavior, and (2) to do so in a low-dimensional representation space, so as to allow effective pattern discovery and learning from experience. The rest of this article offers a brief overview of a comprehensive computational theory that explains how the primate system for visual object recognition solves these two problems. This theory has been implemented and tested both as a computer vision system and as a model of biological vision and is backed by behavioral and neurobiological findings, as detailed in the references.

## 3. A similarity-based framework for visual object processing: the Chorus of Prototypes

In problems that arise in visual object processing (see Table 1), the nature of the stimulus universe and certain generic properties of visual systems ensure that veridical representation of distal object similarities in a low-dimensional space is easy to achieve (for a detailed argument, based on properties of smooth mappings, see Edelman, 1999). In this section, we outline a computational framework that offers a solution to these problems, which is based on the idea of putting similarity itself to work in constructing a representation space for distal objects. Because it represents each stimulus by a vector of its similarities to a small set of reference objects, this framework is called the “Chorus of Prototypes” (Edelman, 1995, 1999).

**Table 1:** **A hierarchy of tasks arising in visual object and scene processing**.

**Task**	**What needs to be done**	**What it takes**
Recognition	Dealing with novel views of shapes	Tolerance to extraneous factors (pose, illumination, etc.)
Categorization	Dealing with novel instances of known categories	Tolerance to within-category differences
Open-ended representation	Dealing with shapes that differ from familiar categories	Representing a novel shape without necessarily categorizing it
Structural analysis	Reasoning about (i) the arrangement of parts in an object; (ii) the arrangement of objects in a scene	Explicit coding of parts and relationships of objects and scenes

The Chorus framework is founded on the observation that, no matter how high-dimensional the measurement space of a visual system is, certain events and relationships of interest “out there” in the world give rise to representational signatures whose structure ensures tractability. One behaviorally important type of such event is the rotation of a rigid object in front of the observer around a fixed axis (or, equivalently, the circumambulation of the object by the observer). Provided that the imaging function that maps the object's geometry into the representation space is smooth, the footprint of the rotation event in the representation space will be a one-dimensional manifold—a smooth curve (which, moreover, will loop back upon itself, due to the cyclic nature of the rotation event)^7^. For rotation around three mutually orthogonal axes, the manifold will be three-dimensional^8^.

### 3.1. Object view spaces

Because the representation of the set of views of a rotating object—its *view space*—has the manifold property, the views can be related to one another by computationally tractable procedures. In particular, given that the view space is smooth, a small number of exemplars (representation-space points that encode particular views of the object) typically suffice to interpolate it, using any of the many existing methods for function approximation. One such method, which, as we shall see in the next section, is especially interesting from the neurobiological standpoint, is approximation by a linear superposition of radial basis functions (Poggio and Edelman, 1990; Poggio and Girosi, 1990).

This corresponds to representing any view of the object by its similarities to a handful of exemplar views that can be learned from experience (Poggio and Edelman, 1990; this, in turn, implies that the view space for the object, as well as a decision function for object identity, can take the form of a weighted sum of the outputs of a set of neurons each of which is broadly tuned to one of the exemplar views). While recognition performance of this mechanism can be highly tolerant to viewpoint changes (if the exemplars are chosen so as to jointly cover the view space well), it is not fully viewpoint-invariant—but neither is the performance of human subjects (Bülthoff and Edelman, 1992; Edelman and Bülthoff, 1992; Edelman, 1999; DiCarlo and Cox, 2007; more about this in section 4).

### 3.2. Object shape spaces

Edelman (1995) noted that the principles that facilitate this kind of low-dimensional representation of relationships between different views of the same object apply also to the relationships between different object shapes. Specifically, object shapes that are not too dissimilar from each other—say, a duck, a goose, and a chicken—can be meaningfully morphed into one another by simple linear interpolation of some fiducial features such as edge configurations, so that intermediate shapes do make sense. Indeed, they form a smooth, low-dimensional manifold.

This implies that under a smooth representation mapping, the set of view spaces of the objects in such a “tight” shape category—its collective *shape space*—can be interpolated by the same means that support the interpolation of individual view spaces (Edelman, 1998). Moreover, because the view spaces of the shapes in question will be roughly parallel to each other, learning a view-related task for one shape would readily transfer to another (Intrator and Edelman, 1996, 1997; Edelman and Duvdevani-Bar, 1997). For instance, learning to predict the appearance of a three quarters view of one face from its frontal view would work also for other faces (Lando and Edelman, 1995; Duvdevani-Bar et al., 1998).

With regards to implementation, the shape space can be approximated by the same means as the view space, as a weighted sum of tuned unit responses, which serve as basis functions. If each of the units is tuned to an entire view space of some object (which may itself appear at a range of orientations), together they will span the shape space for the family of objects in question. Given a potentially novel stimulus, each such tuned unit effectively signals how distant (that is, dissimilar) it is from its preferred shape, or “prototype.” The joint ensemble activity (which inspired the name *Chorus of Prototypes;* Edelman, 1995) pinpoints the location of the stimulus in shape space, just as in a land survey the distances to a handful of landmarks jointly fix the location of a test point in the terrain.

### 3.3. The chorus transform

Formally, representing a new view by its similarities to familiar views or a new shape by its similarities to familiar shapes are both instances of an application of the Chorus Transform (Edelman, 1999). Let **p**_1_,…,**p**_*n*_ be *n* prototypes and let **x** be an input vector, **p**_*k*_, ***x*** ∈ ℝ^d^. The Chorus Transform (CT) is defined as follows:

(1) $$CT\left(x\right)=\frac{1}{\sqrt{n}}\left(\begin{array}{c} \left||x-p_{1}|\right|\\ \vdots \\ \left||x-p_{n}|\right| \end{array}\right)$$

The application of this transform *CT*: ℝ^*d*^ → ℝ^*n*^ results in dimensionality reduction, if the number of prototypical objects, *n*, is smaller than the dimensionality of the measurement space *d*.

Edelman (1999, App.B) showed that the Chorus Transform can support a logarithmic dimensionality reduction, while approximately preserving the inter-point distances in the original space (the proof of this claim is based on a theorem due to Bourgain, 1985). In other words, even with a very small number of prototypes—*O*(log *d*), where *d* is the dimensionality of the original space—the relative positions of the data points in the new, low-dimensional space approximate their original layout, implying that the original similarity relations, and with them category boundaries, etc., are largely preserved^9^.

A statistically robust version of *CT* can be derived by observing that a representation based on distances to a set of points (prototypes) is related to vector quantization (Linde et al., 1980; the following exposition is borrowed from Edelman, 1999, App.B). A vector quantizer *Q* is a mapping from a *d*-dimensional Euclidean space, *S*, into a finite set *C* of *code vectors*, *Q* : *S* → *C*, *C* = (*p*_1_, *p*_2_,…, *p*_*n*_), *p*_*i*_ ∈ *S*, *i* = 1, 2,…,*n*. Every *n*-point vector quantizer partitions *S* into *n* regions, *R*_*i*_ = {*x* ∈ *S* : *Q*(*x*) = *p*_*i*_}; the Voronoi diagram is an example of such a partition. Whereas vector quantization encodes each input pattern in terms of *one* of the code vectors chosen by the nearest-neighbor principle (Cover and Hart, 1967), Chorus does so in terms of similarities to several prototypes. This parallel suggests that a discretized representation of the input space, related to the Voronoi diagram, can be obtained by considering ranks of distances to prototypes, instead of the distances themselves.

Let **p**_1_,…, **p**_*n*_ be *n* prototypes, and consider a representation that associates with each input stimulus the *Rank Order* of its *Distances* to the prototypes (*ROD*). That is, an input **x** is represented by an ordered list of indices *ROD*(**x**) = (*i*_1_, *i*_2_,… *i*_*n*_), meaning that among all prototypes **p**_*i*_, **x** is the most similar to **p**_*i*_1__, then to **p**_*i*_2__, and so on. Note that the index *i* always heads the list *ROD*(**p**_*i*_) corresponding to the prototype **p**_*i*_ (a prototype is most similar to itself). The total number of distinct representations under the *ROD* scheme is *n*! (the number of permutations of the *n* indices). To compare two representations, one may use Spearman rank order correlation of the index lists.

## 4. Experimental support for the chorus framework

The Chorus framework has been implemented and evaluated as a computer vision system for recognition and categorization of isolated objects (Duvdevani-Bar and Edelman, 1999) and for class-based generalization (Lando and Edelman, 1995; Edelman and Duvdevani-Bar, 1997). It had also generated predictions for behavioral, electrophysiological, and imaging experiments, all of which were subsequently corroborated. The relevant studies, which are mentioned briefly in this section, have been discussed at great length elsewhere (Edelman, 1998, 1999).

The basic tenet of the Chorus model—that object vision is fundamentally viewpoint-dependent because its functional building block is a unit broadly tuned to a specific view of a specific object—received early support from psychophysical (Bülthoff and Edelman, 1992; Edelman and Bülthoff, 1992) and neurophysio-logical (Logothetis et al., 1994; Logothetis and Pauls, 1995; Wachsmuth et al., 1994; Perrett and Oram, 1998) experiments. Subsequent studies consolidated the notion that object recognition is characterized not by invariance but by tolerance to extraneous factors such as orientation and retinal position, which, furthermore, depends on the task and on the prior experience with the objects in question (Dill and Edelman, 2001; DiCarlo and Maunsell, 2003; Cox et al., 2005; Rust and DiCarlo, 2010).

A particularly interesting feature of the Chorus framework is that object representations that it posits are *generically veridical* with regard to inter-object similarities. As noted above, the dimensionality reduction method employed by the Chorus model—representing each stimulus by its distances to shape-space landmarks—is guaranteed to approximately preserve original similarities among stimulus shapes, insofar as it implements the random subspace projection method of near-isomorphic embedding (Johnson and Lindenstrauss, 1984; Bourgain, 1985). The predicted metrically veridical perception of object similarities has indeed been demonstrated in behavioral and physiological studies with humans (Cutzu and Edelman, 1996, 1998; Edelman et al., 1998, 1999; Giese et al., 2008; Panis et al., 2008) and monkeys (Sugihara et al., 1998; Op de Beeck et al., 2001).

In summary, results from human and monkey psychophysics and physiology suggest, as predicted by the Chorus framework, (1) that the visual system seeks tolerance rather than invariance to object transformations (Rust and DiCarlo, 2010), as predicted by the view- and shape-space idea (Edelman et al., 1998; DiCarlo and Cox, 2007), (2) that object translation can be disruptive, especially for structure representation (Dill and Edelman, 2001; Cox et al., 2005; Kravitz et al., 2008), as predicted by the retinotopy of the classical receptive fields that are the functional building blocks of the Chorus model, (3) that this trait is compatible with extrastriate neural response properties (Vogels, 1999; Gallant et al., 2000; DiCarlo and Maunsell, 2003), and (4) that the peculiarities in the manner in which primate vision deals with object structure (Tsunoda et al., 2001; Newell et al., 2005; van Dam and Hommel, 2010) can be accounted for by a fragment-based scheme that relies on binding by retinotopy Edelman and Intrator (2003).

## 5. A renewed interest in the mathematics of similarity and the chorus transform

The past decade saw a variety of new and exciting developments in the theory of similarity-preserving associative recall, which are proving to be widely useful in computer vision, notably LSH (Andoni and Indyk, 2008). Furthermore, some old ideas for embedding structured data in vector spaces, such as holographic reduced representations (Plate, 1991), are being rediscovered and applied (Jones and Mewhort, 2007), albeit not in the visual domain. We see both these sets of development as important to visual scene representation and processing: the former contribute to the struggle against the curse of dimensionality, while the latter suggest computationally convenient and neurally plausible ways of dealing with structure. In this section and in section 6, we briefly describe representative methods from these two domains and show that they are either related to the Chorus Transform or can benefit from its application.

### 5.1. The chorus transform implements locality-sensitive hashing (LSH)

Significant progress in similarity-based high-dimensional data management has been recently brought about by the development of new algorithms that perform hashing while respecting local similarity (Andoni and Indyk, 2008; Paulevé et al., 2010). The growing family of *LSH* algorithms “effectively enables the reduction of the approximate nearest neighbor problem for worst-case data to the exact nearest neighbor problem over random (or pseudorandom) point configuration in low-dimensional spaces” (Andoni and Indyk, 2008). Both steps in this process—forming the random projections and quantizing the resulting low-dimensional space into address bins—rely on the same computational principles that underlies the Chorus Transform and can be carried out by the same mechanism, namely, a set of tuned units.

**Figure 1:**
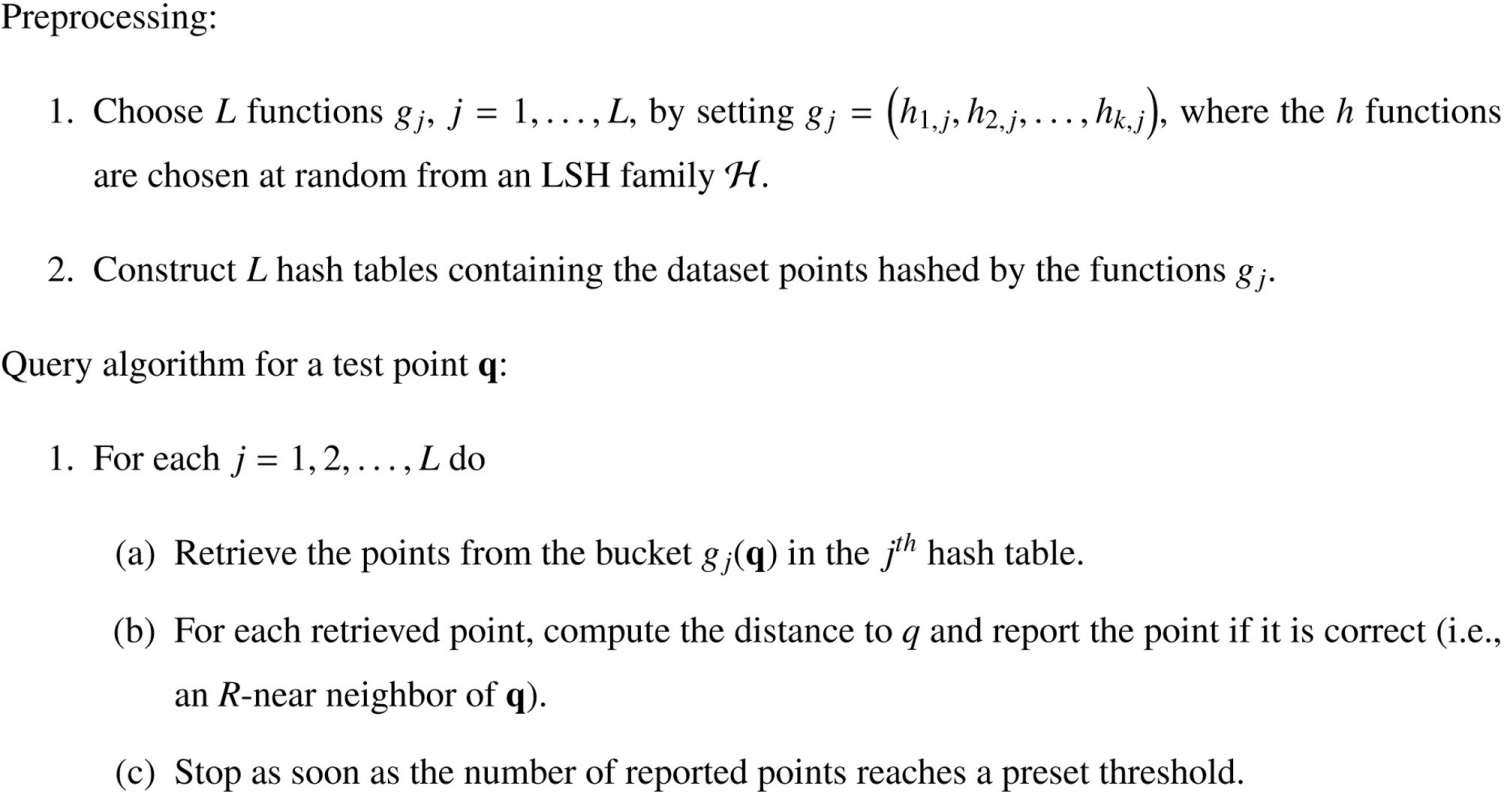
**The locality-sensitive hashing (LSH) scheme (after Andoni and Indyk, 2008, Figure 2).** For an explanation of how the Chorus Transform implements LSH, see section 5.1.

As outlined in Figure 1, the process begins by choosing a number of hash functions from a family of functions ℋ = {*h*: ℝ^*d*^ → *U*} that satisfies the LSH condition: the probability *P*_1_ of mapping two data points **p**, **q** ∈ ℝ^*d*^ to the same bin must be larger than the probability *P*_2_ of mapping them to different bins if the points are close together —

(2) $$\text{if}||p-q||\leq R\;\;\text{then}\;{\text{Pr}}_{ℋ}\left[h\left(p\right)=h\left(q\right)\right]\geq p_{1}$$

(3) $$\text{if}||p-q||\geq cR\;\;\text{then}\;{\text{Pr}}_{ℋ}\left[h\left(p\right)=h\left(q\right)\right]\leq p_{2}$$

where *R* is the radius of the neighborhood that defines proximity and *c* > 1 is a constant (which defines an “exclusion zone” around the *R*-neighborhood). Each of the hash functions is then used to construct a hash table, which are populated by points from the given data-set. The lookup procedure for a query point **q** iterates over the hash tables and returns retrieved points that fall within an *R*-neighborhood of **q**.

Now, consider the “multidimensional line partitioning” LSH family described by Andoni and Indyk (2008, p. 121). A hash function from this family first performs a random projection of the data point **p** into ℝ^*t*^, where *t* is super-constant [i.e., grows slowly with *n*, as in *t* = *o*(log *n*)]. The space ℝ^*t*^ is then partitioned into cells, and the hash function is made to return the index of the cell that contains the projected point **p**.

This last part suggests a ready parallel to the Chorus Transform. Specifically, the receptive fields of the tuned units representing the prototypes effectively function as the cells in the second step of the above procedure (the first step being the projection of the probe point on the manifold defined implicitly by the choice of prototypes). To complete the analogy, the outputs of the tuned units can be thresholded (as in the *ROD* version of the transform), so that the resulting code consists of the identities (that is, indices) of units whose activation by the probe point exceeds the threshold.

The original Chorus Transform, without thresholding, can be seen to carry out *kernelized* LSH (a variant introduced by Kulis and Grauman (2009), which, as those authors note, is applicable to both vector and non-vector data). In a recent development of this approach, He et al. (2010, p.1133) defined the space *V*_*j*_ onto which the data are projected by the *j*^*th*^ hashing function by a linear combination of “landmarks” {**z**_*n*_} in the kernel space. This idea leads to the hash function.

(4) $$h\left(p\right)=sign\left(a^{T}k_{p}-b\right)$$

where **a** are the linear combination weights and

(5) $$k_{x}={\left[K\left(x,z_{1}\right),\ldots ,K\left(x,z_{n}\right)\right]}^{T}$$

are the kernel values between **x** and each of the landmark points **z**_*n*_. With the distance function || · || serving as the kernel and **z**_*n*_ as the prototypes, this corresponds precisely to an application of the Chorus Transform to the data point **x**.

### 5.2. The chorus transform computes concomitant statistics

In their discussion of LSH families, Andoni and Indyk (2008, p. 120) note that if the Jaccard similarity, defined for two sets *A* and *B* as *s*(*A, B*) = |*A* ∩ *B*|/|*A* ∪ *B*|, is used as a basis for hashing, the LSH framework is thereby extended to include the so-called *minwise hashing* methods. Minwise hashing (Broder, 1997; Li and König, 2011) is a special case of pairwise characterization of ordered sets through their concomitant statistics (Eshghi and Rajaram, 2008, Section 4), and is best explained as such.

Consider *n* independent sample pairs, {(*x*_1_, *y*_1_), (*x*_2_, *y*_2_),…, (*x*_*n*_, *y*_*n*_)} obtained from a bivariate distribution *f*(*x, y*). In the theory of rank order statistics, *y*_*k*_ is called the *concomitant* of *x*_*k*_. Formally, concomitant theory captures the relation between the order statistics of *x* and *y* in the form of a rank distribution given by Pr[*Rank*(*y*_*i*_) = *j* | *Rank*(*x*_*i*_) = *k*].

Let ∏^*n*^_1,1_ be the probability that the smallest of *x*_*i*_ is the concomitant of the smallest of *y*_*i*_. The link to the LSH theory now becomes apparent: if the smallest element among *x*_*i*_ is identical to that of *y*_*i*_, it must lie in the intersection of the two sets, which implies that the probability ∏^*n*^_1,1_ is equal to the Jaccard similarity between them (this is the defining insight behind minwise hashing, due to Broder, 1997).

Eshghi and Rajaram (2008) observe that the same reasoning holds not just for the smallest (lowest-ranking) pair but also for any range of smallest concomitant ranking pairs of the two sets. They proceed to define a “min *k*-multi-hash” LSH family based on this observation. For us, it is of interest because the smallest *k* values in a Chorus Transform—a representation that supports LSH—are effectively computed by retaining the smallest *k* out of the *n* distances to the prototypes that define it^10^.

In a related vein, Yagnik et al. (2011) introduce the Winner Take All (WTA) hash, “a sparse embedding method that transforms the input feature space into binary codes such that Hamming distance in the resulting space closely correlates with rank similarity measures.” Their hash functions define the similarity between two points by the degree to which their feature dimension rankings agree. Yagnik et al. (2011) point out that the simplest of such measures is the pairwise order function *PO*(*x, y*) = ∑_*i*_ ∑_*j* < *i*_ *T*((*x*_*i*_ − *x*_*j*_) (*y*_*i*_ − *y*_*j*_)), where *x*_*i*_ and *y*_*i*_ are the *i*^*th*^ dimension values of **x**, **y** ∈ ℝ^*n*^ and *T* is a threshold function, *T*(*x*) = 1 if *x* > 0 and *T*(*x*) = 0 otherwise.

Whereas Yagnik et al. (2011) proceed to define their WTA hash family using random permutations of feature dimensions, it can also be formulated in terms of the Chorus Transform. To that end, in lieu of permuting the dimensions, all we have to do is administer a vector of random biases (drawn from a predetermined set of random vectors) to the landmark units; each such bias vector effectively permutes the rank order of the unit responses. Given that under the Chorus Transform, the output representation by distances to prototypes preserves the rank order of data point similarities in the original space (Edelman, 1999, App.B), the above procedure is exactly equivalent to the one proposed by Yagnik et al. (2011), with the added advantage of being carried out in a more convenient low-dimensional space.

## 6. Extending the chorus framework to cover structural similarity

**Figure 2:**
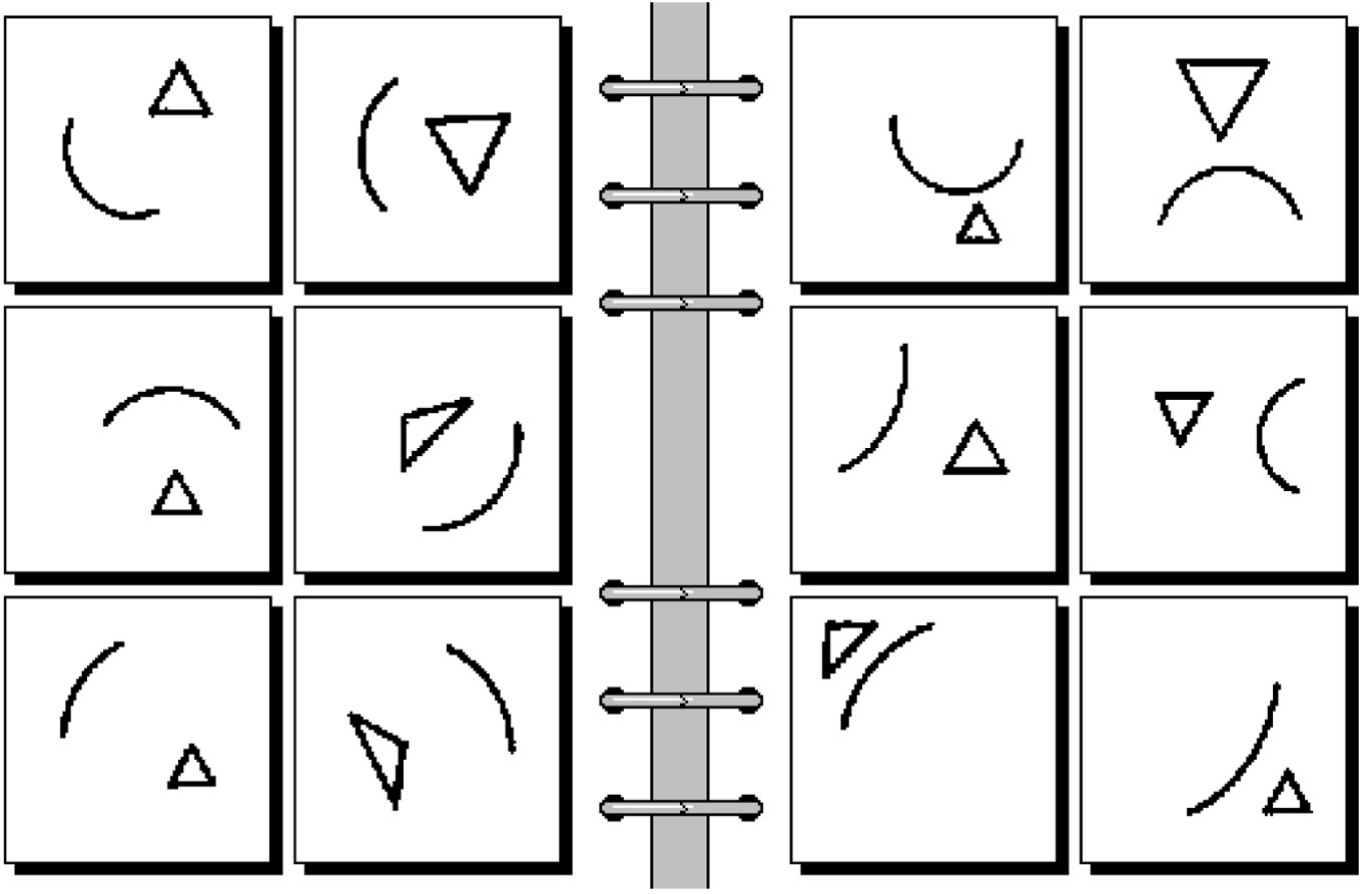
**Problem #75 of the 100-long sequence of challenges to pattern recognition posed by Bongard (1970).** The task is to determine what distinguishes the scenes on the left from the scenes on the right. To answer this question, it is not enough to list the shapes that appear in the scenes: their spatial attitudes and relations must be made explicit too. This representational requirement is often referred to as (a spatial counterpart to) structural *systematicity* (Edelman and Intrator, 2003). See text for discussion.

The kinds of visual stimuli discussed up to now in this paper did not include objects composed of parts or scenes containing multiple objects, such as those depicted in Figure 2, or that which you will see if you raise your eyes from this paragraph and look around you. In this section we first list some of the functional requirements posed by structured scenes and the challenges presented by those requirements. We then briefly mention a previously published biologically motivated model of scene processing (Edelman and Intrator, 2003). Finally, we outline a new computational approach to scene interpretation, the Chorus of Relational Descriptors (ChoRD), which uses *CT* on all the representational levels: for representing shapes, their relationships, and entire scenes.

### 6.1. Functional requirements and challenges in composite scene interpretation: systematicity and structural alignment

Operational parsimony, which in animal vision translates into evolutionary pressure, dictates that a visual system should represent a structured scene hierarchically, in terms of intermediate-size parts and their spatial relations, if such a representation is warranted for the family of scenes at hand by the MDL principle (Rissanen, 1987; Adriaans and Vitányi, 2007). Ideally, therefore, the representation of scene structure would be fully compositional in the classical sense of Frege (1891)^11^.

A compositional representation would allow the visual system to be *systematic* in its interpretation of parts and relations—a desideratum that is traditionally invoked in support of compositional models based on MDL (Bienenstock et al., 1997). Formally, an agent employing symbolic representations is systematic if its ability to entertain the proposition *R*(*a, b*) implies a concomitant ability to entertain the proposition *R*(*b, a*). In vision, this would mean that a system that can make sense of a scene in which a man rides a donkey should also be able to make sense of a scene in which a donkey rides a man (Edelman and Intrator, 2003, Figure 1). In practice, however, human cognition is often far from systematic in its dealing with structure, and so is unlikely to rely on fully compositional representations (see Johnson, 2004 for informal arguments and Edelman and Intrator, 2003 for empirical evidence).

**Figure 3:**
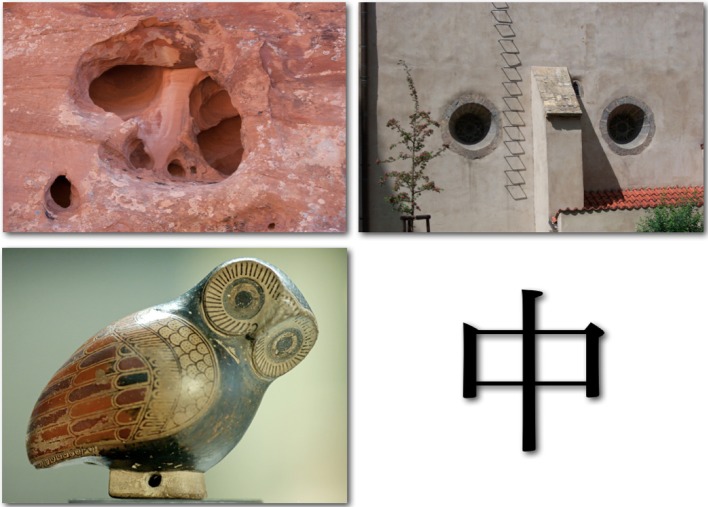
**Four scenes for which possibilities for structural alignment can be profitably explored.** Image sources: *top left*, a pattern in weathered sandstone, Lower Muley Twist Canyon, Capitol Reef National Park, Utah; *top right*, the eastern wall of the Old Synagogue, Jewish Quarter, Prague; *bottom left*, a proto-Corinthian figurine of an owl, ca. 640 B.C. (from the antiquities collection at the Louvre); *bottom right*, the Chinese character for “middle” *(zhōng)*.

If a modicum of systematicity is to be preserved, a certain amount of spatial analysis must be carried out (Edelman and Intrator, 2003), so as to enable *structural alignment* (Markman and Gentner, 1993)—a procedure in which parts and relations found in one scene are matched to parts and relations found in the other^12^. Consider, for instance, the two scenes at the top of Figure 3. Disparate as these scenes are, certain parallels can be drawn between some fragments of one and fragments of the other. In particular, the vertical ridge at the center of the sandstone depression in the scene on the left resembles the narrow vertical lean-to attached to the wall of the building depicted in the scene on the right. Furthermore, each of the two circular windows on both sides of this vertical feature can be matched, respectively, to two rounded (but not very circular) holes in the scene on the left. In each of the two scenes, the spatial arrangement of the matched fragments forms a stylized face (two eyes and a nose between them)—a realization that in turn suggests structural similarity to the spatial composition of the head of the owl in the scene on the bottom left and, stretching the imagination a bit, to the Chinese character on the bottom right of Figure 3.

Structural alignment thus turns the question of scene interpretation (and with it also the question of scene similarity) into a nested set of questions about similarities of scene parts and their relations. The four scenes resemble each other (up to a point) *because* each one consists of individually alignable fragments (the “eyes” and the “nose”) that, moreover, form the same spatial pattern on a larger scale. Given a proper interpretation of each of those scenes, we can answer questions such as “what shape appears to the left of the vertical feature?”, “what feature appears between the rounded ones?” or “what is the structural counterpart of *this* vertical feature in the other scene?”

What kind of representation can meet these functional needs without running afoul of constraints imposed by neural implementation? Let us suppose for the moment that the representations of structured objects or scenes are themselves made to possess an analogous symbolic structure. Following this logic, the representation of a scene composed of two shapes, one above the other, could take the form of an ordered pair of the two feature vectors corresponding to the two constituent shapes. This approach, however, creates a dilemma. On the one hand, it relies on abstract relational binding (which is how the ordered pairing of constituents is implemented in symbolic models; see, e.g., Hummel and Holyoak, 1998; Hummel, 2001). Although such an implementation, being fully compositional, would result in ideal systematicity, it is not, we believe, entirely biologically or behaviorally plausible, as noted above^13^. On the other hand, eschewing symbolic binding in favor of a more biologically relevant approach, such as representing composite scenes by bags of features each of which carries both shape and location information (cf. the “what + where” features of Rao et al., 1997; see also Op de Beeck and Vogels, 2000) has problems of its own in supporting structural alignment, insofar as scene constituents are not easy to address selectively in such a representation.

### 6.2. An early approach: the chorus of fragments

Edelman and Intrator (2000; 2003) attempted to avoid both horns of the above dilemma by developing the Chorus of Prototypes into a non-compositional model of structure representation that exhibits appropriately limited systematicity. Instead of positing generic parts and abstract relations, their *Chorus of Fragments* model relied on the scene layout and on binding by retinotopy to represent structure and on multiple location-bound shape spaces to represent its constituents. The resulting model exhibited a degree of systematicity, in that it interpreted correctly spatial rearrangements of shapes familiar to it through training (namely, digit shapes). It also showed productivity, in that it performed nearly equally well for novel shapes, which had had no “what” units dedicated to them (letter shapes).

The model, described in detail by Edelman and Intrator (2003), consisted of “what + where” units, which by definition respond selectively in a graded manner both to stimulus shape and to its location (Rao et al., 1997; Op de Beeck and Vogels, 2000). During learning, it relied on multiple fixations to train the functional equivalent of a shape-tuned (“what”) unit parameterized by location (“where”). This functionality, which can be thought of as gain modulation through covert attention shifts (Connor et al., 1997; Salinas and Abbott, 1997; Salinas and Thier, 2000), offers a solution of sorts to the problem of constituent addressing, which, as we just mentioned, arises in structural alignment. During testing, a single fixation of the composite stimulus by the model sufficed for interpreting it—that is, for making explicit, through the pattern of the units' responses, of what shape was present at what location in the stimulus.

### 6.3. A new idea: chorus of relational descriptors (ChoRD)

While the CoF model did the right thing in predicating a full representation of a scene on multiple fixations of its constituents, it implemented the “what + where” functionality using a black-box learning mechanism (a bottleneck autoencoder; DeMers and Cottrell, 1993) that performed the task while leaving its inner workings opaque. In this section, we describe a new approach to implementing limited systematicity and thereby supporting various structure-related tasks, which is characterized by two main features. First, similar, to the CoF model, it is constrained by the architectural and functional considerations that call for distributed, graded, low-dimensional representations. Second, it improves on the CoF model by dealing explicitly with the many related versions of the same scene arising from multiple fixations, and by doing so through recourse to the same computational mechanism that is at the core of *CT*: representation by similarities to multiple prototypes. Because of that, the new approach has also the advantage of being related to the similarity-preserving hashing methods that are being currently used in computer vision (as we pointed out in preceding sections).

The new approach, Chorus of Relational Descriptors, or ChoRD, represents a given scene by multiple entries in an associative memory. The memory system is implemented by a hash table of the LSH type, in which (1) each of the possibly many entries for a given scene uses one of the scene's regions of interest (ROIs) as the key, and (2) key values falling within a certain range of similarity to a given ROI are all mapped to the same record. The record associated with a key ROI is the scene minus that ROI; it is represented by a list of the remaining ROIs along with the spatial displacement of each of them relative to the key ROI.

To give a concrete example, consider a scene consisting of an object, **A**, which appears *above* another object, **B** (in general, of course, a scene can consist of more than two objects). Representations of this scene will be stored in the hash table under two keys, *ROI*(**A**) and *ROI*(**B**)—and so will scenes that contain objects sufficiently similar to **A** and **B**. In particular, the representation stored under *ROI*(**A**) will consist of the list {*ROI* (**B**), dir (**A, B**)}, where the last element encodes the direction from **A** to **B**.

The ChoRD model that we just outlined uses *CT* on two levels. First, and most fundamentally, both the ROIs comprising the scene and their relative spatial displacements with regard to each other are represented by vectors of distances to select sets of shape and layout prototypes, respectively. Second, given that an LSH-based representation is itself equivalent to *CT* (as we showed in section 5.1), the entire scene is de facto represented in a distributed, redundant, graded fashion by the ensemble of records associated with its constituent ROIs, in a manner that neither discards the spatial structure of the scene, nor attempts to capture it categorically, as the symbolic models aim to do.

## 7. Testing a simple implementation of ChoRD

We now describe a series of tests of the ChoRD model, carried out in the simple domain of scenes composed of two ROIs each (a detailed examination of the model's performance and its scaling to more complex scenes will be reported elsewhere; Shahbazi and Edelman, in preparation). Each scene was constructed by embedding two object images, drawn from six most populous object categories in the LabelMe database (Russell et al., 2008), in a black background. The objects were converted to grayscale and scaled to a size of 50 × 50 pixels; the entire scene was 150 × 150 pixels (see Figure 7 for some scene examples). While this type of test image will probably fail to impress computer vision practitioners, it has the advantage of allowing a very tight control over the scene parameters, which is why such scenes are at present widely used in behavioral and imaging studies (e.g., Newell et al., 2005; Hayworth et al., 2011; MacEvoy and Epstein, 2011; Zhang et al., 2011), some of whose results we replicate below.

### 7.1. Encoding the ROIs and their layout

**Figure 4:**
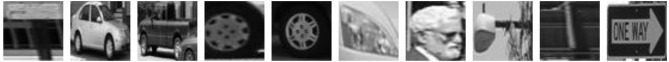
**The 10 shape prototypes used in conjunction with *CT* to encode the ROIs comprising the scenes (see section 7.1).** Each ROI detected in a scene was represented by a 10-dimensional vector of its respective similarities to these 10 images.

Regions of interest (ROIs) were detected in the scene by sliding a Gaussian patch along the image and locating the ROI at the place that resulted in a maximum sum of the pixel values of the convolved image. The size of the Gaussian patch was made to match the size of the objects. Ten objects were chosen at random from the list of LabelMe objects to serve as the prototypes for *CT* (see Figure 4). Each of those was represented by a list of outputs of Gabor filters at two different scales, 5 and 10 pixels, and two orientations, 0° and 90°^14^. Every detected ROI patch was represented by the list of filter values, then encoded by the 10-prototype *CT*.

**Figure 5:**
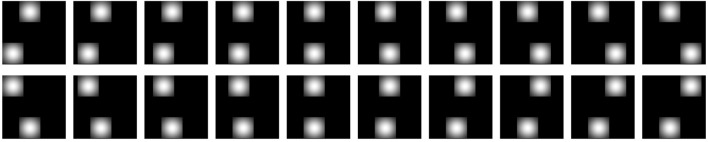
**The layout prototypes used in conjunction with *CT* to encode the spatial structure of scenes (see section 7.1).** There are two different sets of such prototypes. One set of 10 prototypes is used for encoding the scene when the top ROI is fixated; the other set of 10 prototypes is used when the bottom ROI is fixated. For each situation (scene + fixation), the scene structure was thus represented by a 10-dimensional vector of similarities between the layout of the scene's ROIs and the 10 layout prototypes.

To encode the spatial structure or layout of the scene, we represented it by similarities to a set of 10 layout prototypes. Fixation-dependent encoding was simulated by using one such set of 10 layouts for cases in which the top ROI was fixated and another one for cases in which the bottom ROI was fixated (see Figure 5). Each layout prototype consisted of two Gaussian image patches. The image location of one of these, corresponding to the would-be scene placement of the reference or key ROI for the given fixation, was fixed, and the location of the other differed systematically among the 10 prototypes, spanning collectively a range of displacements as illustrated in Figure 5. The entire scene's layout was therefore encoded relative to the fixation point (the location of the key ROI) by listing its image-based similarities to the 10 displacement prototypes.

**Figure 6:**
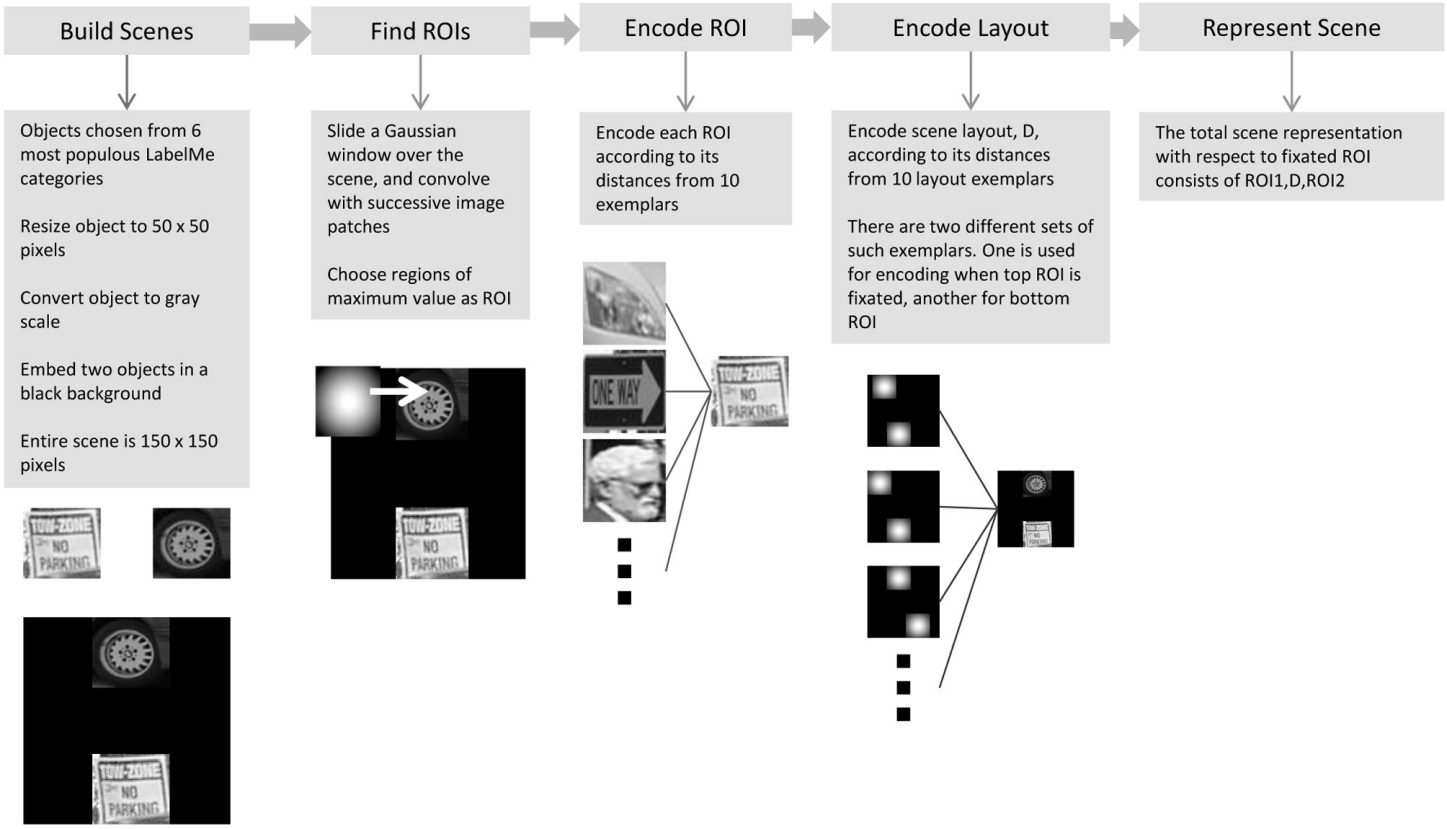
**The procedure for computing a ChoRD representation of a scene.** The representation of each encountered scene is entered into the model's LSH table, with the representation of the fixated ROI serving as the key. See text for additional details.

The entire procedure whereby the representation of a scene was computed is illustrated in Figure 6. Altogether, the complete representation of a scene for a given fixation (“entry” or key) point consisted of the concatenation of (1) a 10-dimensional representation of the fixation ROI, (2) a 10-dimensional representation of the other ROI, and (3) a 10-dimensional representation of the spatial layout relative to fixation. Scene representations constructed in this manner were entered into an LSH table, implemented using Shakhnarovich's Matlab code with ten 64-bit hash tables (Shakhnarovich, 2008).

The LSH functionality (which, as we showed in section 6, is equivalent to that of *CT*) subsequently allowed content-based lookup—a key ingredient in testing the resulting ChoRD model on additional scenes, which could be familiar or novel in some respects. In the experiments described in the remainder of this section, we tested the ability of the ChoRD model to support certain systematicity-related queries and to replicate several behavioral and imaging studies involving human subjects.

Following training (that is, populating the LSH with scene representations), each familiar scene is represented redundantly, by as many records as it has ROIs. Given a test scene, the model's LSH table returns all the representations that match the ROIs contained in it. Importantly, because of the locality-sensitive property of the hashing scheme that we used, a novel scene—that is, a scene that differs somewhat from the familiar examples either in its ROIs or in their locations, or both—results in the retrieval of familiar scenes that are sufficiently similar to it. Thus, we expected the model's performance to degrade gracefully when tested on progressively more novel stimuli, rather than crash.

### 7.2. Experiment 1: productivity

Our first experiment tested the model's productivity: its ability to deal with moderate novelty as just defined. Each of the test stimuli in this experiment had one novel and one familiar object in a familiar configuration, two novel objects in a familiar configuration, or two familiar objects in a novel configuration. The dissimilarity between the test scene and the representation retrieved in response to it was defined as

(6) $$\Delta_{k}=||ROI_{11}-ROI_{12}||+||D_{11}-D_{12}||+||ROI_{21}-ROI_{22}||$$

where *ROI*_*ij*_ is the *i*^*th*^ *ROI* of scene *j*, and *D*_*ij*_, is the layout representation of scene *j* relative to *ROI*_*ij*_. Identical computations were performed by fixating each of the two objects in the test scene, yielding Δ_1_ and Δ_2_, which were then averaged together to form the composite dissimilarity between the two scenes.

We remark that the form of Eq. 6 glosses over the conceptual difficulty inherent in trying to deal simultaneously with multiple shape and location differences. This difficulty is universal in that it arises in any attempt to compare composite entities (say, estimating the similarity of two sets of fruit containing one apple and one orange each), including certain structural alignment tasks (section 6.1). In psychology, this corresponds to the classical problem of scaling (Shepard, 1987), which is beyond the scope of the present discussion. Thankfully, in the present context of *testing* a given model (rather than defining the representation that serves as its foundation), this difficulty amounts merely to a matter of preference that may or may not be given to some components of the composite dissimilarity, depending on the task. This can be done simply by weighting those components as needed. Our choice in Equation 6 corresponds to using equal weights for all.

**Figure 7:**
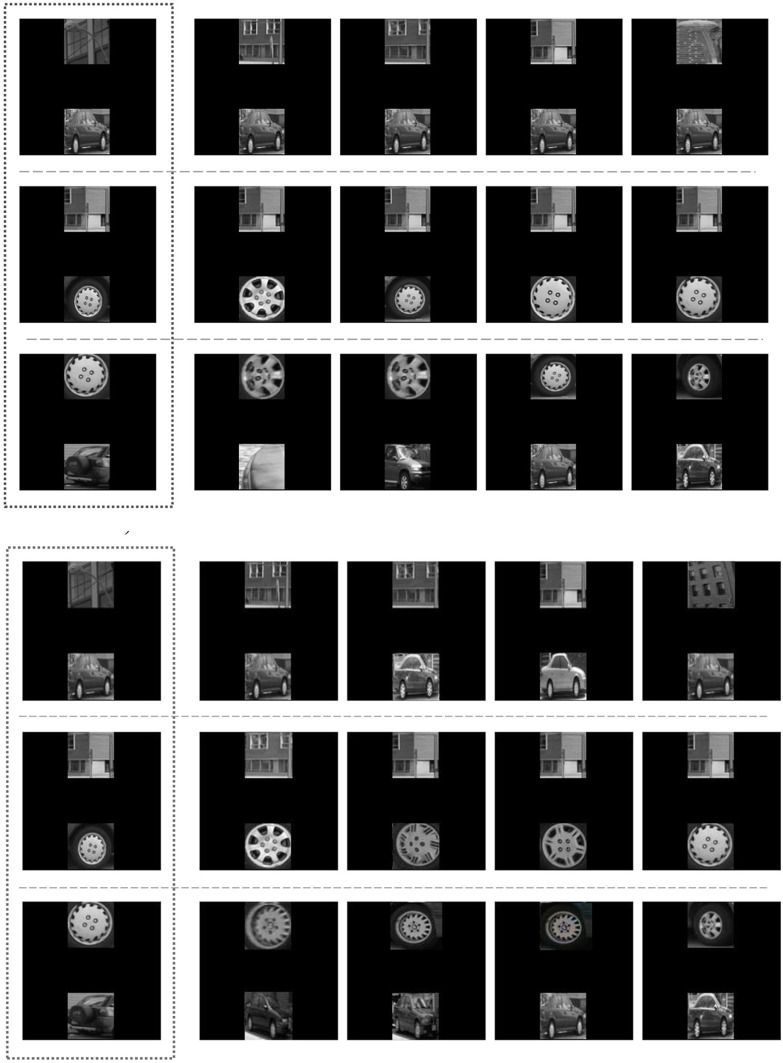
**Experiment 1, testing productivity.** See section 7.2 for a description of the procedure and Figure 8 for quantitative results. *Above:* the performance of the ChoRD model, which uses *CT* to represent ROIs. The leftmost column shows test scenes; the other columns show the best five matches retrieved from the model's LSH table, in the decreasing order of similarity to the test scene. **Top row**: One novel object at position *ROI*_1_. **Middle row**: One novel object at position *ROI*_2_. **Bottom row**: Two novel objects. *Below:* the performance of a version of the model that uses raw ROI encoding rather than one based on *CT* (the layout was still encoded with *CT*).

The experiment was performed on 6000 test scenes in three different conditions: condition N, 2000 test scenes with one novel object; condition NN, 2000 test scenes with two novel objects; and condition L with 2000 test scenes with two familiar objects in a new spatial layout. For each condition, the test scene was encoded according to both possible fixations, and the query was performed for both encodings. For each query, the five nearest neighbors were retrieved and their (dis)similarity to the test scene was computed. The reported results are for the best match obtained (i.e., the most similar scene retrieved from the hash table). Figure 7 shows examples of test scenes (on the left) and their corresponding five most similar scenes retrieved from the table.

**Figure 8:**
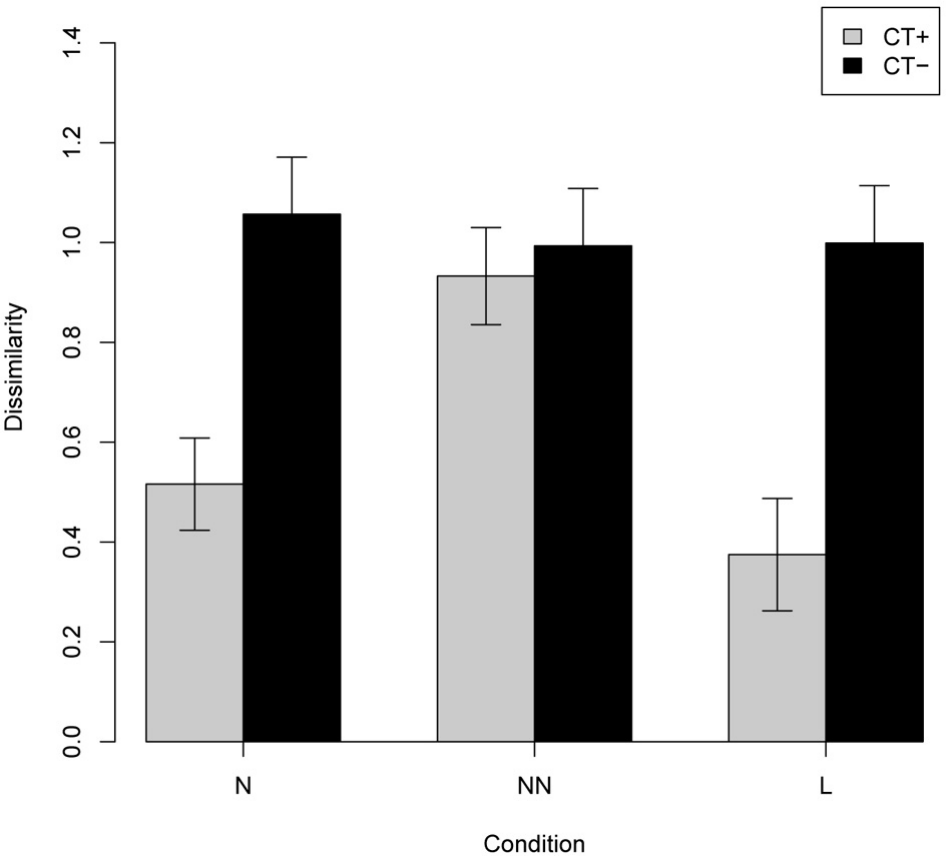
**Productivity as measured by dissimilarity between test and retrieved scenes (experiment 1; means with 95% confidence intervals) of the full version of the ChoRD model, which uses *CT* both for ROI and for layout representation (gray bars) compared to that of a version that uses raw ROI encoding (black bars).** Cf. Figure 7, top and bottom, respectively.

To investigate the contribution of *CT* to the model's performance, we carried out another experiment, this time using the raw filter-based encoding of the scenes. Figure 8 shows side by side the results for the raw and *CT*-encoded scenes. Note that there is no significant difference in the similarity of the test and retrieved scenes for different conditions in the non-CT version.

### 7.3. Experiment 2: sensitivity to gradual change

**Figure 9:**
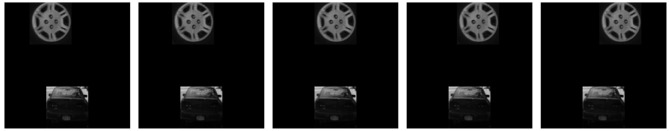
**An example of five scenes used in one trial of experiment 2 (sensitivity to gradual changes; see section 7.3).** In each image, *ROI*_1_ is displaced by 10 pixels relative to the scenes on either side.

**Figure 10:**
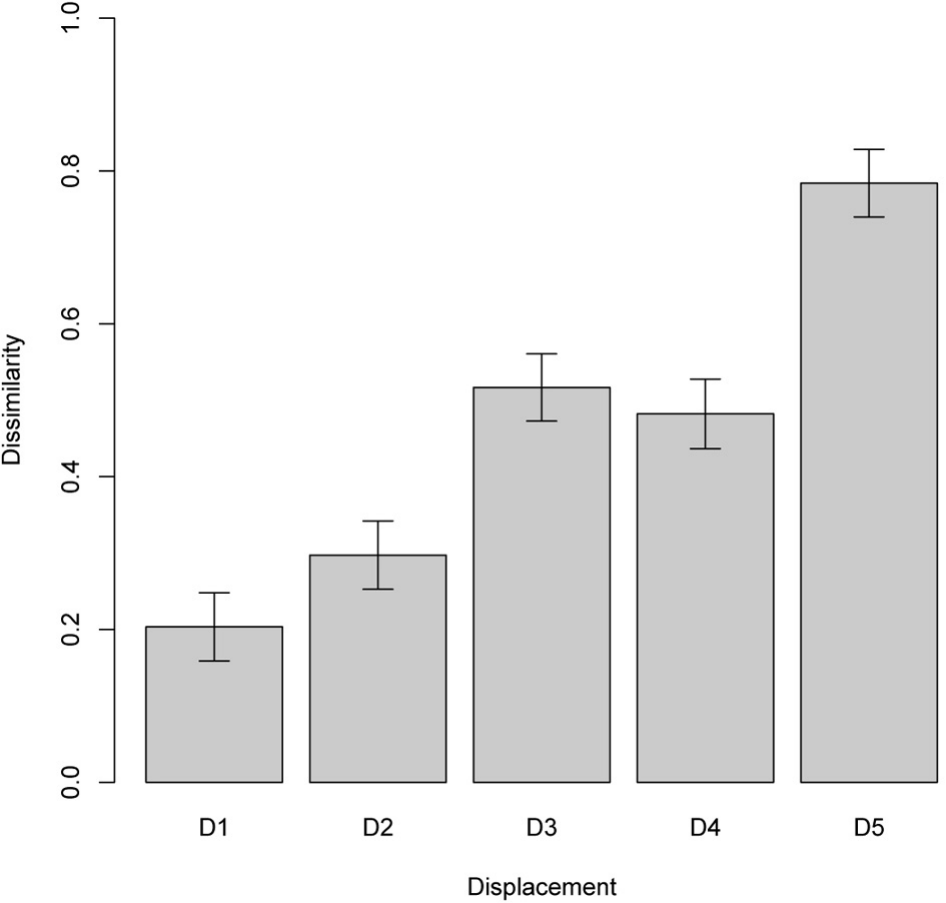
**Experiment 2, sensitivity to gradual change.** The plot shows the difference between two scenes composed of the same objects (means with 95% confidence intervals), with different amounts of displacements: 10, 20, 30, 40, and 50 pixels. The pattern of results replicates that of the corresponding experiment in (Newell et al., 2005).

In the second experiment, we measured the similarity of two scenes represented by the ChoRD model, in one of which the two objects were progressively displaced relative to each other (see Figure 9). Newell et al. (2005) found that the performance of human subjects in this situation indicated their reliance on representations that yielded graded similarity, rather than breaking down categorically as the layout of the manipulated scene changed. To simulate their study, we generated a series of test scenes with the same two objects. By keeping one object's position constant and displacing the other one, the relative positions of the objects were changed, either horizontally or vertically, in increments of 10 pixels. Figure 10 shows the resulting dissimilarities between reference and test scenes. The experiment was performed on 2000 different scenes, with five levels of displacement tested for each scene, and resulted in a gradual increase of dissimilarity with displacement. A linear regression fit the results well: *R*^2^ = 0.72, *F*_(9998)_ = 2.06 × 10^4^ (*p* < 2.2 × 10^−16^).

### 7.4. Experiment 3: sensitivity to different types of qualitative change

Our third experiment examined the ChoRD model's representation of relative similarities of scenes that were subjected to certain structural transformations. It has been patterned on the imaging study of Hayworth et al. (2011), who showed that for human subjects the BOLD response of brain areas implicated in scene representation is more sensitive to some structural transformations than to others. In particular, for scenes composed of two objects, switching the two objects around resulted in a larger release of adaptation, compared to simply translating both objects within the scene while keeping their relative positions unchanged.

**Figure 11:**
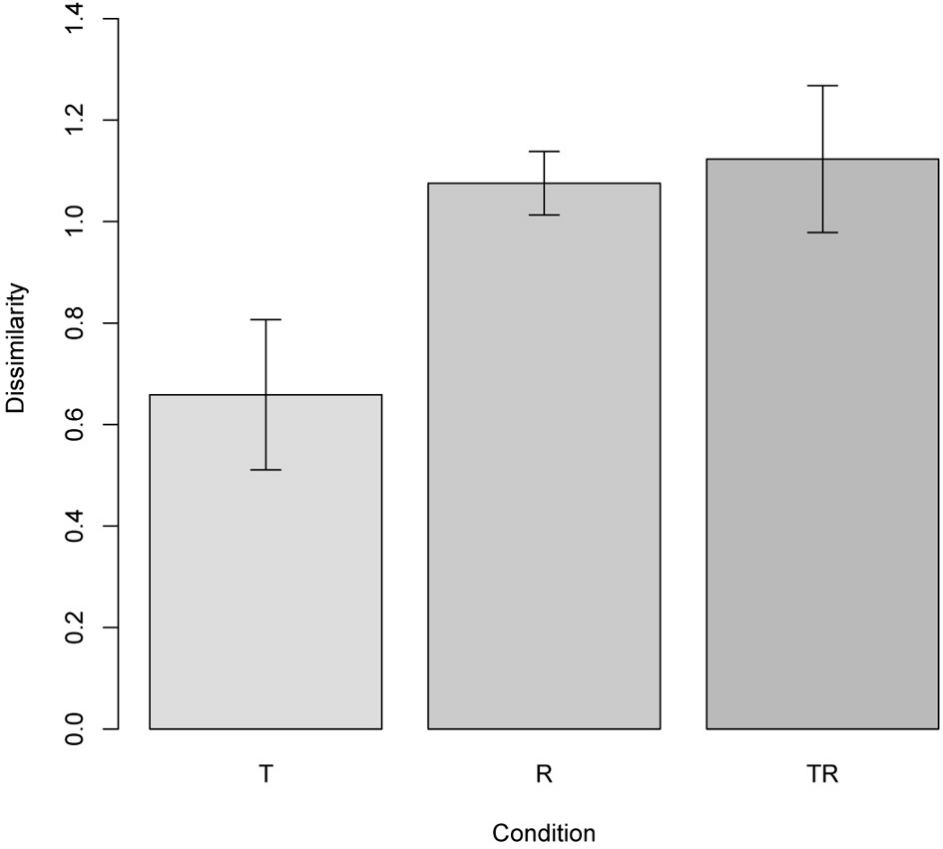
**Experiment 3, sensitivity to qualitative change; see section 7.4.** The plot shows the difference between two scenes (means with 95% confidence intervals), one of which has been generated from the other via three types of structural transformations: translation (T), reversal, or switching the two objects around (R), and both translation and reversal combined (TR). The results replicate those of the corresponding experiment in (Hayworth et al., 2011).

To replicate this finding, we constructed test scenes related to reference ones in three ways: through a joint translation of both objects (condition T), or reversal of the objects' locations (condition R), or both (condition TR). Two thousand scenes were generated for each of these conditions. The results, plotted in Figure 11, conform to those of Hayworth et al. (2011).

### 7.5. The ChoRD model: a discussion

We have tested the ChoRD model on simple scenes composed of two objects, in three experiments. In the first experiment, the model exhibited a degree of productivity, that is, an ability to deal, systematically, with scenes that differed in various ways from those to which it had been exposed during “training” (cf. Edelman and Intrator, 2003). In the second experiment, we found that the model's estimate of similarity between a reference scene and a series of test scenes differing from it progressively was it self graded—a finding that echoed that of Newell et al. (2005) in a similar setup. In the third experiment, we used the model to replicate one of the findings of an fMRI adaptation study (Hayworth et al., 2011), which found differential effects on brain activation of two types of scene transformation: joint translation vs. switching around of the scene's constituents. All these results were obtained by a model that used *CT* on every relevant representational level to reduce dimensionality and enact tolerance to moderate novelty, supporting our assertion of the importance of similarity-based representations in scene processing.

In addition to being rooted in our own earlier work on similarity-based object and scene representation (Edelman, 1999; Edelman et al., 2002; Edelman and Intrator, 2003), the ChoRD model can be seen as related to several contemporary lines of thinking in computer vision, as mentioned very briefly below (a detailed comparison will be offered in Shahbazi and Edelman, in preparation). In particular, the location-specific *CT*-based representations used here resemble the locality-constrained linear coding of Wang et al. (2010). The relationship between *CT* and vector quantization (VQ), from which Wang et al. (2010) derive their approach, has been noted and analyzed in (Edelman, 1999; cf. section 3.3). Continuing this parallel, the graded manner in which *CT* codes the similarities between the target object and prototype shapes may be compared to the variant of VQ that uses soft assignment (van Gemert et al., 2010).

Whereas many computer vision methods for image representation and retrieval rely on the bag of (visual) words idea (which goes back to the first histogram-based approaches developed two decades ago), there is an increasing number of attempts to extend this simple and powerful principle to capture some of the scene structure (and not just the mere presence in it of certain objects). One step in this direction is expressed by the “context challenge” of Torralba (2003), which led to the development of such successful systems for context-based recognition as that of Divvala et al. (2009). Our model can be seen to engage with this challenge by coding scenes relative to certain “entry points” or key objects, for which the rest of the scene then constitutes a context (of course, it still needs to be tested in an actual context-based recognition task).

We single out the work of Zhang et al. (2011) on image retrieval using geometry-preserving visual phrases (GVP) as the closest to ChoRD among the present computer vision approaches. Rather than trying to make scene structure matter by subjecting a set of images, preselected on the basis of bag of visual words similarity, to a spatial voting test (RANSAC; Fischler and Bolles, 1981), Zhang et al. (2011) incorporate information about relative spatial locations of the features forming a visual phrase into its representation (hence “geometry-preserving”). Compared to GVP, the ChoRD model appears to be more flexible and open-ended, insofar as it relies on *CT* in representing both the features and their layout.

Insofar as the ChoRD model represents a scene by a set of records keyed to its constituents and stored in an LSH table, it can be said to treat a scene merely as a big object. Imaging evidence for this kind of scene representation in the lateral occipital complex in the human brain has been reported recently by MacEvoy and Epstein (2011), who write that “patterns of activity evoked in LO by scenes are well predicted by linear combinations of the patterns evoked by their constituent objects.” Notably, there was no evidence of such summation in the parahippocampal place area (PPA), implicated by previous studies in the representation of scene structure (Epstein and Kanwisher, 1998; Bar, 2004). In comparison, in the ChoRD model, the spatial structure of the scene is not lost in summation, as it would be under a bag of features approach. This pattern of results suggests to us the following tentative double analogy: (1) between the (distributed, *CT*-based) ChoRD representation of constituent shape and the LO complex, and (2) between the (also *CT*-based) ChoRD representation of scene layout and the PPA.

## 8. Conclusions

In the first part of this paper, we surveyed the role of similarity in theories and models of object recognition and described some newly discovered computational parallels between the Chorus Transform, or *CT* (an idea that received a book-length treatment in Edelman, 1999) and the widely popular computer vision methods of similarity-preserving hashing and dimensionality reduction. In the second part, we described the outcome of some (rather preliminary) tests of the ChoRD model, which extends *CT* so as to support a joint representation of scene content and layout. In this concluding section, we outline some of the directions in which the similarity project can be extended.

Taken together, our findings suggest that similarity to prototypes may constitute a viable general approach to representing structured objects and scenes. In particular, the same *CT*-based method can be used to span view spaces of individual shapes and shape spaces of object categories (Edelman, 1999), as well as “scene spaces” defined by objects and their spatial relations (the present work). From the computational standpoint, this is an exciting development, given that scene-related work in computer vision tended until recently to focus on scene categorization rather than interpretation (Oliva and Torralba, 2001; Lazebnik et al., 2006; Loeff and Farhadi, 2008).

The approach proposed here can support scene interpretation (over and above categorization), insofar as a list of objects, contexts, and relations to which a given scene is similar constitutes a rather complete representation of its content and structure (just like in a text local adjacency relations within character n-grams jointly enforce global structure of phrases; cf. Wickelgren, 1969; Mel and Fiser, 2000). In computer vision, similar ideas underlie the work on “visual phrases” (Sadeghi and Farhadi, 2011; Zhang et al., 2011) and Conditional Random Fields (Kulkarni et al., 2011, Figure 3). To ensure flexibility, this representation should be parameterized by task, so that the similarity patterns revealed by it could focus on shape similarity (say) in some cases and on spatial relation similarity in others; a related idea has been proposed by Edelman and Intrator (2003, Figures 6 and 7).

We believe that further development of the similarity-based representational framework outlined in this paper should focus on the following three issues.

**Neural implementation.** Edelman and Intrator (2003) discussed the biological plausibility of their similarity-based scheme that coded scene fragments and their spatial relations (which they called the Chorus of Fragments). Indeed, this approach seems quite amenable to a neural implementation: a set of laterally interacting receptive fields, each tuned to an object category and embedded in a retinotopic map, would seem to do the job. More thought needs, however, to be given to the implementation of tuning. In particular, units that employ radial basis functions are not good at rejecting false positives. This calls for alternatives such as Exemplar-SVM (Malisiewicz et al., 2011), which may, perhaps, be amenable to implementation by augmenting RBF units with massive inhibition (Wang et al., 2000).

**Scalability.** Much progress has been achieved in computer vision by methods that utilize huge databases of images (e.g., Malisiewicz and Efros, 2009). Given the close relationship between the Chorus framework and similarity-tolerant hashing, which we detailed in section 5, those methods may be on a convergence course with our approach. This may in turn result in a biologically inspired emulation of the vast human memory for visual objects and scenes (e.g., Brady et al., 2008).

**A probabilistic turn.** The Chorus framework is deterministic in its operation, its only stochastic aspect being the choice of prototypes during learning; it is also purely feedforward. While such models may be adequate for categorization tasks (Serre et al., 2008), they do not allow for the kind of flexibility that is afforded by the generative Bayesian approach (Tenenbaum and Griffiths, 2001; Chater et al., 2006). It is often the case, however, that successful models of learning and inference can be recast in Bayesian terms with very little modification (Edelman and Shahbazi, 2011). Developing the Chorus framework into a hierarchical generative model^15^ is, therefore, a worthwhile future pursuit, which may take as its starting points the use of maximum-entropy reasoning and the Bayes theorem by Shepard (1987) and the generative theory of similarity proposed by Kemp et al. (2005).

In summary, we remark that the idea that similarity could play a key explanatory role in vision (as well as in other cognitive sciences) has experienced ups and downs in the centuries since its introduction by Hume. The Chorus project has previously shown that coding objects by their similarities to select prototypes can support a veridical representation of distal similarities among objects “out there” in the world, and to do so in a low-dimensional space that affords effective learning from experience. The ChoRD approach to representing structure enables the extension of the Chorus framework to composite objects and scenes. Moreover, the deep parallels between the Chorus idea and similarity-preserving hashing techniques indicate that the resulting methods could be made to scale up to deal with massive amounts of visual data. These developments suggest that vision researchers would do well to renew their respect for similarity and assign it a key role in their conceptual toolkit.

### Conflict of interest statement

The authors declare that the research was conducted in the absence of any commercial or financial relationships that could be construed as a potential conflict of interest.

## Footnotes

^1^We discuss a similarity-based approach to dealing with the effects of orientation and juxtaposition of objects in scenes later in this paper. For related approaches to countering the effect of illumination, which rely on similarity to previously encountered exemplars, see for instance (Shashua, 1992; Sali and Ullman, 1998). Evidence that the human visual system relies on prior experience in its treatment of illumination in face recognition is offered by Moses et al. (1996).

^2^Cf. Shepard's (1987) notion of consequential regions, and the need for differential valuation of stimulus dimensions implied by the Ugly Duckling Theorem (Watanabe, 1969, pp. 376–377).

^3^This observation applies to natural or analog similarities, not symbolic or conventional ones. Thus, a heap of 19 marbles is naturally similar to a heap of 20 marbles under any of a wide range of visual measurement schemes, whereas under most schemes the number 19 on this page is only conventionally similar to the number 20. A natural similarity space for shapes is discussed in (Edelman, 1999, 3.2–3.3).

^4^Note that this formulation is related to the more general view of the problem of learning from examples as the estimation of the joint probability density over input and output variables.

^5^The support vector approach to supervised learning can solve classification and regression tasks directly in a high-dimensional space; see Cortes and Vapnik (1995) for an early formulation and Malisiewicz et al. (2011) for a recent application.

^6^Despite its intuitive appeal and deep roots that go back to Plato, the first-order isomorphism approach is also infeasible in practice (given the computational difficulties associated with the task of reconstructing the world from sensory data) and is a poor model of human performance (given that subjects are in fact very bad at such reconstruction).

^7^For definitions of formal concepts such as smoothness and manifolds, and for other mathematical details, see Edelman (1999).

^8^If the object is opaque, the manifold will be piecewise smooth.

^9^Recent developments in neighborhood-preserving embedding and immersion (Bartal et al., 2011) improve on the Johnson and Lindenstrauss (1984) result that had been cited by Edelman (1999). The original J-L lemma states that any *n*-point subset of Euclidean space can be embedded in *O*(ϵ^−2^ log *n*) dimensions with at most (1 + ϵ) distortion of the inter-point distances. In contrast, the new *local dimension reduction* lemma (Bartal et al., 2011) offers a likewise bounded-distortion embedding into a space whose dimensionality does not depend on *n*, as long as it is the local and not the global structure of the data set that is to be preserved. It remains to be seen whether this embedding method can be carried out by mechanisms whose biological implementation is as straightforward as that of the Chorus scheme.

^10^These are the *k* landmarks that are the closest to the probe data point; cf. the discussion of the relationship between *CT* and vector quantization in section 3.3. We also note that this idea is related to the coding scheme of Thorpe et al. (1996) and the MAX model of Rousselet et al. (2003).

^11^For a thorough introduction to the principle of compositionality, see (Szabó, 2008); for a discussion in the context of vision, see (Edelman and Intrator, 2003).

^12^Structural alignment differs from shape alignment for recognition, introduced by Huttenlocher and Ullman (1987) and Ullman (1989), in that it operates on the objects' parts (which, further, could be defined in terms of their function rather than shape) and relations, instead of on the global shapes of the objects.

^13^Concerns about biological plausibility arise also with regard to the otherwise fascinating idea of representing structured objects in the same metric space as simple ones, as in the Holographic Reduced Representations of Plate (1991) and other approaches based on similar mathematical principles (e.g., Jones and Mewhort, 2007; Sahlgren et al., 2008; Basile et al., 2011).

^14^The original implementation of *CT*-based object recognition (Duvdevani-Bar and Edelman, 1999) used an even simpler ROI representation with great effect. In a modern computer vision setting, a SIFT-based representation (Lowe, 1999) would be used.

^15^The importance of hierarchy in this context is underscored by the recent finding that human observers learn to interpret hierarchically structured scenes more readily than others (Shahbazi et al., 2011).
